# Quality assessment of available Internet information on early orthodontic treatment

**DOI:** 10.1186/s12903-024-04019-w

**Published:** 2024-03-19

**Authors:** Mehmed Taha Alpaydin, Tugce Alpaydin, Merve Koklu, Suleyman Kutalmış Buyuk

**Affiliations:** Department of Orthodontics, Faculty of Dentistry, Ordu University, Ordu, 52200 Turkey

**Keywords:** Early orthodontic treatment, Internet, Patient information, Quality of information, Websites analysis

## Abstract

**Background:**

This study aimed to evaluate the content, reliability, quality and readability of information on Internet websites about early orthodontic treatment.

**Methods:**

The “early orthodontic treatment” search term was individually entered into four web search engines. The content quality and reliability were reviewed with DISCERN, Journal of American Medical Association (JAMA), and Health on the Net code (HONcode) tools using the contents of websites meeting predetermined criteria. The readability of websites was evaluated with Flesch Reading Facilitate Score (FRES) and Flesch–Kincaid Grade Level (FKGL).

**Results:**

Eighty-six websites were suitable for inclusion and scoring of the 200 websites. 80.2% of websites belonged to orthodontists, 15.1% to multidisciplinary dental clinics and 4.7% to professional organizations. The mean DISCERN score of all websites (parts 1 and 2) was 27.98/75, ranging between 19 and 67. Professional organization websites had the highest scores for DISCERN criteria. Moreover, 45.3% of websites were compatible with JAMA’s disclosure criterion, 7% with the currency criterion, 5.8% with the authorship criterion and 5.8% with the attribution criterion. Only three websites met all JAMA criteria, and these websites belonged to professional organizations. None of the websites had the HONcode logo. Mean FRES and FKGL were 47.6 and 11.6, respectively.

**Conclusions:**

The quality of web-based information about early orthodontic treatment is poor, and readability is insufficient. More accurate and higher quality Internet sources are required on the web.

## Introduction

Early orthodontic treatment is described as an type of orthodontic treatment performed during the deciduous and early mixed dentition period [1, 2]. The purpose of early orthodontic treatment is to correct or prevent further development of malocclusions in this period and facilitate possible orthodontic treatment in permanent dentition [3]. Any abnormal deformity and pathology intervening in the normal development of occlusion should be eliminated or minimized with preventive orthodontic treatment. In addition, early orthodontic treatment could decrease the complexity of future malocclusion [4–6].

The Internet has been used increasingly in the fields of communication, education, shopping and healthcare [7, 8]. This increase in the use of individuals on Internet has had a great impact on access to online health information, and 70%of adult individuals in the United States make Google search every year [9]. The Internet has the characteristic of being an information archive independent of its usage purposes. The abundance of information on the Internet creates virtual information pollution in the online environment, making it difficult for individuals to access accurate and reliable information. This situation necessitates the evaluation of the accuracy and reliability of information [10]. Thus, many evaluation tools have been developed to evaluate websites in many aspects [11–13]. Evaluation of the readability of Internet-based health information is very important because the difficulty of readability limits the usability of websites [14, 15]. The difficulty of reading online health articles on websites is much higher than recommended [16].

Many studies have been performed on the relevant websites’ information resources and content quality on many topics related to orthognathic surgery, orthodontic pain, lingual orthodontics and orthodontics [11, 17–20]. However, no studies investigated website content quality regarding early orthodontic treatment. Therefore, this study aimed to evaluate the quality and readability of the information provided by websites on early orthodontic treatments.

## Materials and methods

Google Trends [21] (Alphabet Inc, Mountview, Calif, USA) was used to determine the key term among the two related terms and “early orthodontic treatment” and “interceptive orthodontics” terms were compared. It was found that the “early orthodontic treatment” term has been the most used term by Google users in the United States to search websites within the last five years. “Early orthodontic treatment” term was searched in 4 web search engines commonly used in the United States [Google (Google LLC, Mountain View, CA, USA), Bing (Microsoft, Redmond, WA, USA), DuckDuckGo (DuckDuckGo, Inc, Paoli, Pennsylvania) and Yahoo (Yahoo, Sunnyvale, CA, USA)] [22]. The search was performed on October 8, 2022, by a single user (M.K.) using a virtual private network (VPN) in the United States. VPN extends a private network over a public network and allows users to send and receive data shared by users as if in a private network. Because the authors of this manuscript do not live in the United States, VPN was used to simulate the virtual environment of the United States. The key term was searched in each search engine and the first 50 websites were recorded. Only English websites were analyzed. Duplicate websites, advertisements, links to scientific articles, videos, social media profiles, forums, blogs, discussion groups and unrelated websites were excluded. The websites were categorized according to authorship or ownership for further analysis.

DISCERN index, Journal of the American Medical Association (JAMA) comparison criteria and Health on the Net code (HONcode, Health on the Net Foundation, Geneva, Switzerland) were used to evaluate the contents of websites in terms of quality and reliability [11–13, 23]. DISCERN index is the first standard index used by consumers to evaluate the quality of health-related information. Index consists of 3 parts and 16 questions scored from 1 to 5. One point signifies “totally disagree” and 5 points signify “totally agree”. Part 1 consists of 8 questions and signifies the reliability of the article. Part 2 consists of 7 questions and analyzes the quality of treatment options. Part 3 consists of 1 question about the overall quality of the website (Table 1). DISCERN website [12], contains information about what we should consider while deciding on the score of each question. Websites were evaluated within the specified ranges except for the 16th question (range 15–75; 15–26: Very poor, 27–38: Poor, 39–50: Average, 51–62: Good, and 63–75: Excellent).

**Table 1:** The questions of the DISCERN instrument with the mean (standard deviation) score of each question

Each question is rated accordingly:						Orthodontists	Multidisciplinary dental clinics	Professional organizations	Total
	1	2	3	4	5				
	No		Partially		Yes	**Mean (SD)**	**Mean (SD)**	**Mean (SD)**	**Mean (SD)**
**Section 1 (Questions 1–8)**: ***The reliability of the publication***									
**1.** Are the aims clear?						2.28 (0.48)	2.62 (0.65)	3.70 (1.06)	4.00 (0.82)
**2.** Does it achieve its aims?						2.19 (0.49)	2.38 (0.51)	3.70 (1.06)	4.00 (0.82)
**3.** Is it relevant?						2.13 (0.38)	2.46 (0.66)	3.70 (1.06)	4.00 (0.82)
**4.** Is it clear what sources of information were used to compile the publication (other than author/ producer)?						2.10 (0.79)	1.69 (0.48)	2.90 (1.45)	4.00 (2.00)
**5.** Is it clear when the information used or reported in the publication was produced?						1.00 (0.00)	1.00 (0.00)	2.70 (1.64)	5.00 (0.00)
**6.** Is it balanced or unbiased?						3.78 (0.59)	3.69 (0.63)	3.50 (1.27)	3.50 (1.91)
**7.** Does it provide details of additional sources of support and information?						1.04 (0.36)	1.00 (0.00)	3.00 (1.63)	3.75 (1.89)
**8.** Does it refer to areas of uncertainty?						1.29 (0.57)	1.46 (0.66)	2.90 (1.45)	3.25 (0.96)
**Section 2 (Questions 9–15)**: ***The quality of the information on treatment choices***									
**9.** Does it describe how each treatment works?						1.31 (0.53)	1.54 (0.66)	3.30 (1.16)	2.75 (1.50)
**10.** Does it describe the benefits of each treatment?						1.94 (0.38)	1.92 (0.87)	3.50 (0.85)	2.00 (1.15)
**11.** Does it describe the risks of each treatment?						1.03 (0.17)	1.15 (0.53)	2.70 (1.16)	2.00 (1.15)
**12.** Does it describe what would happen if no treatment is used?						2.62 (0.60)	2.23 (0.73)	1.30 (0.67)	2.50 (1.29)
**13.** Does it describe how treatment choices affect the overall quality of life?						1.07 (0.26)	1.31 (0.48)	3.50 (0.71)	2.50 (1.73)
**14.** Is it clear that there may be more than one possible choice of treatment?						1.14 (0.55)	1.62 (0.87)	3.60 (1.35)	3.00 (1.41)
**15.** Does it provide support for shared decision-making?						2.10 (0.42)	2.31 (0.63)	3.10 (1.29)	3.00 (0.82)
**Section 3 (Question 16)**: ***Overall quality rating of the publication***									
**16.** This question is rated accordingly:									
	**1**	**2**	**3**	**4**	**5**	2.16 (0.41)	2.31 (0.48)	3.20 (0.92)	3.50 (0.58)
	Low Serious or extensive shortcomings		Moderate Potentially important but no serious shortcomings		High Minimal shortcomings				

JAMA comparison criteria were used to determine the reliability and acceptability of medical information on websites. Websites were evaluated as authorship (authors, contributors, links), attribution (references and sources for the content and copyright information), disclosure (potential conflict), and currency (dates of submission and update) [13].

The websites were evaluated for the HONcode logo. HONcode is the oldest and most trusted logo to evaluate the quality of medical information on the Internet. HONcode evaluates websites containing health-related information over eight objective criteria. HONcode certificate is given to websites that meet these criteria for one year, and yearly evaluations are performed [23].

The readability of websites is evaluated with Flesch Reading Ease Score (FRES) and Flesch-Kincaid Grade Level (FKGL) scores. FRES evaluates websites in terms of readability, with a score ranging from 0 to 100. Scores 90–100, 80–89, 70–79, 60–69, 50–59, 30–49, and 0–29 were classified as very easy, easy, fairly easy, standard, fairly hard, hard and very hard, respectively. Scores were automatically calculated with an online FRES calculator using approximately 300 terms on each website [24]. FKGL scores were also calculated with the same calculator. FKGL provides scores corresponding to the United States education grade level to understand the website information.

All data were analyzed using a statistical analysis program (SPSS® Inc., Windows version 26; IBM, Armonk, NY, USA). Data were expressed as mean (±), standard deviation (SD), median (minimum-maximum) and frequencies (percentage). The Shapiro-Wilk test was employed to evaluate the normal distribution of the data. The test results indicated that the data did not follow a normal distribution. Consequently, non-parametric tests were conducted. Mann-Whitney *U* test was used for intergroup comparisons to analyze the effect of authorship. Fisher’s Exact test was used to compare JAMA criteria between groups.

All included websites were re-evaluated two weeks later by the same author (M.K.) with DISCERN and JAMA scores. Also, the intraexaminer was evaluated with an intra-class correlation coefficient (ICC). The ICC scores of DISCERN and JAMA were 0.942 and 1.000, respectively and showed excellent intra-class correlation coefficients.

## Results

**Fig. 1:**
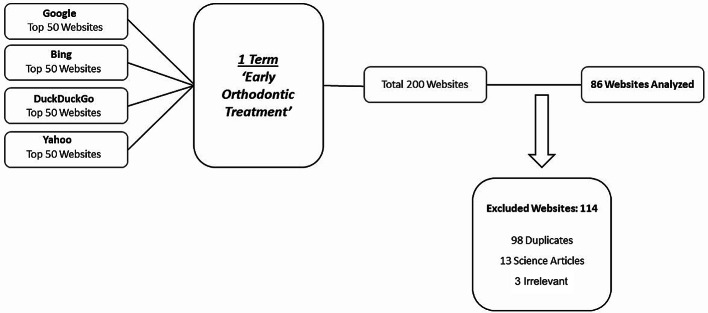
Flowchart diagram of Internet search

After applying inclusion and exclusion criteria, 86 websites were evaluated (Fig. 1). The websites were categorized according to ownership and authorship. Most websites (*n* = 69; 80.2%) were created by orthodontists. This group was followed by multidisciplinary dental clinics (*n* = 13; 15.1%). Only 4.7% of the websites (*n* = 4) belonged to professional organizations, including orthodontics societies and online general health information websites. The number of websites belonging to professional organizations included in the study was not significant and therefore not included in the statistics. None of the websites had the HONcode logo.

**Fig. 2:**
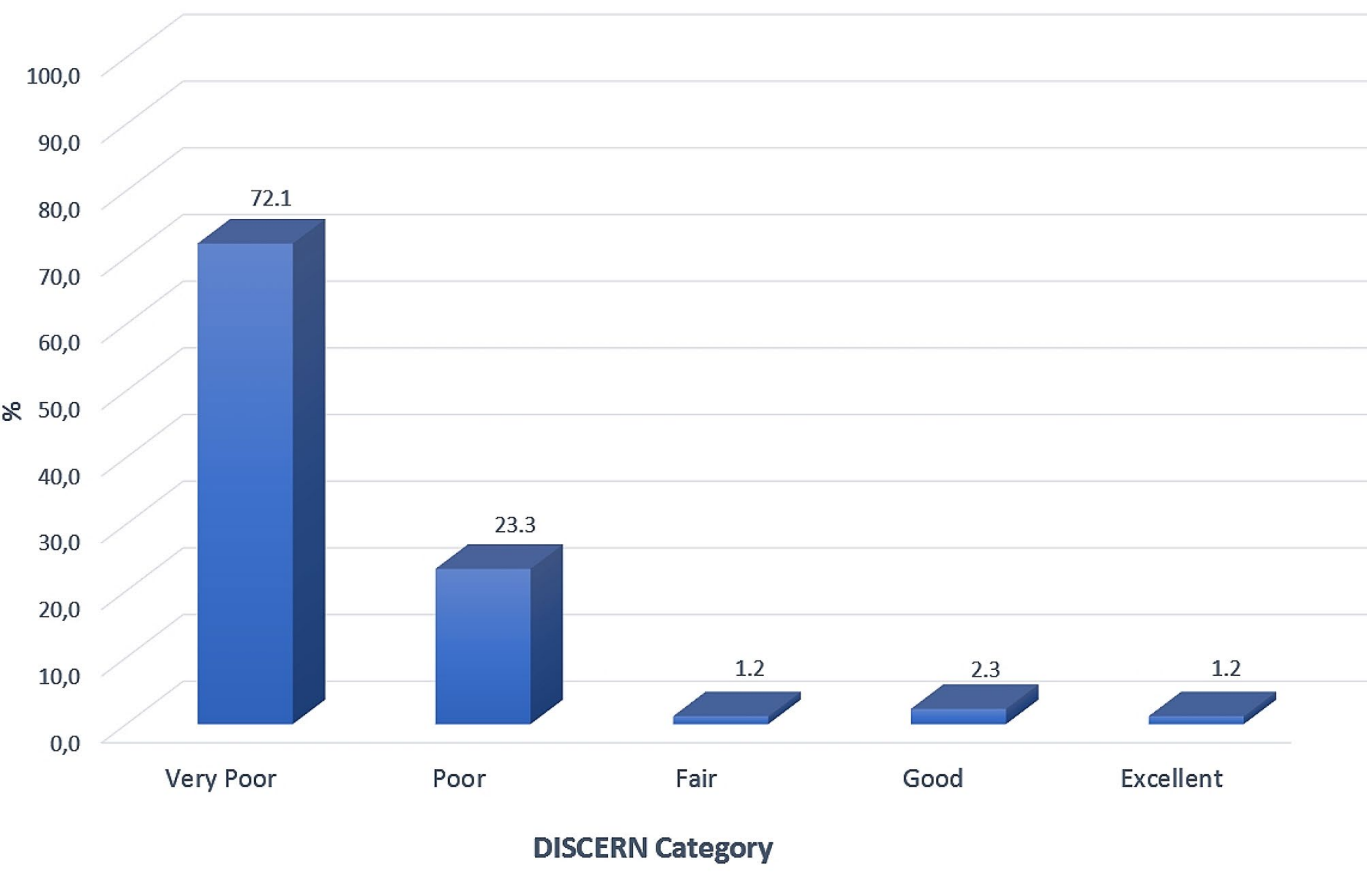
Distribution of DISCERN scores of analyzed websites

**Fig. 3:**
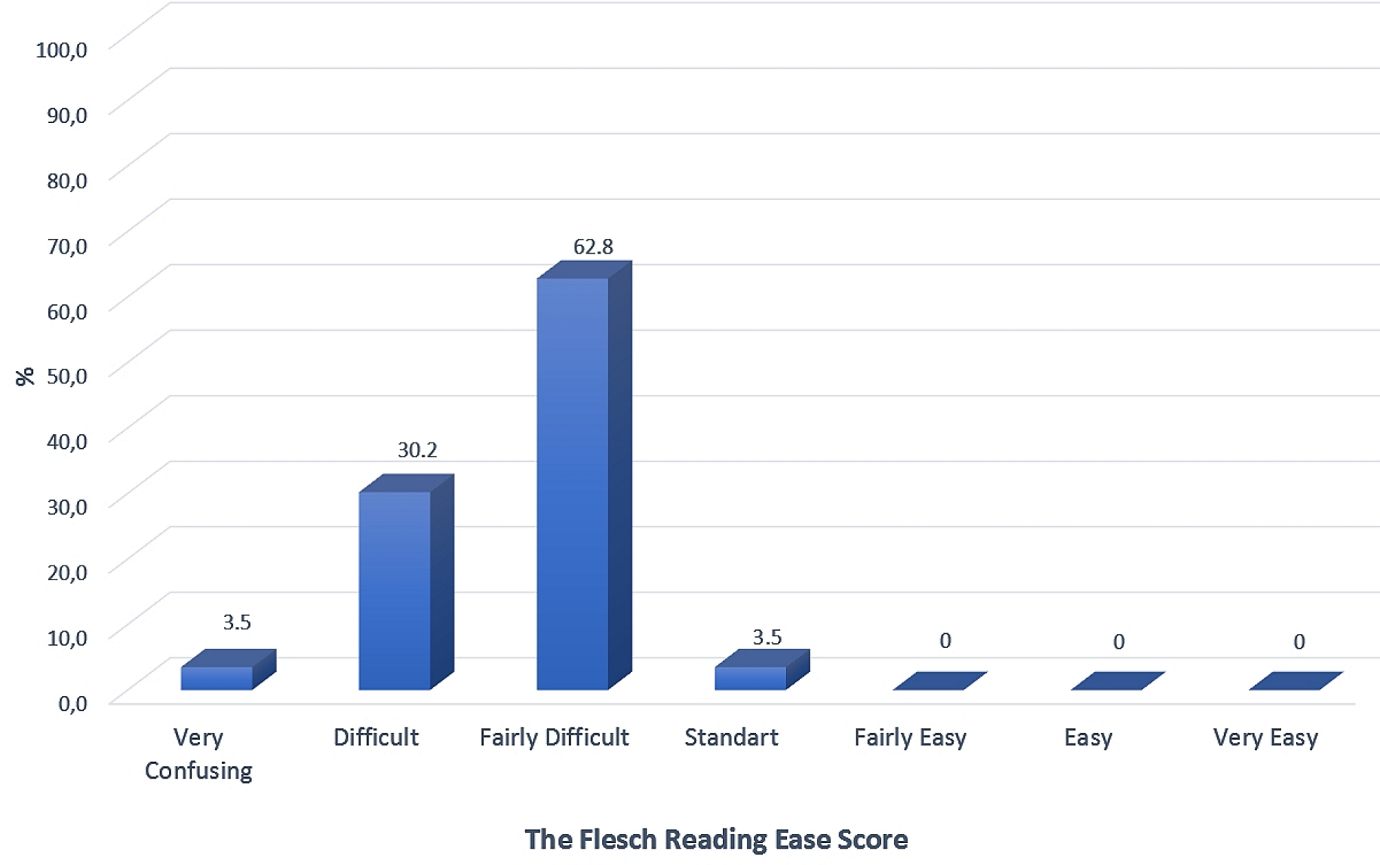
Flesch reading ease score (FRES) of analyzed websites

**Fig. 4:**
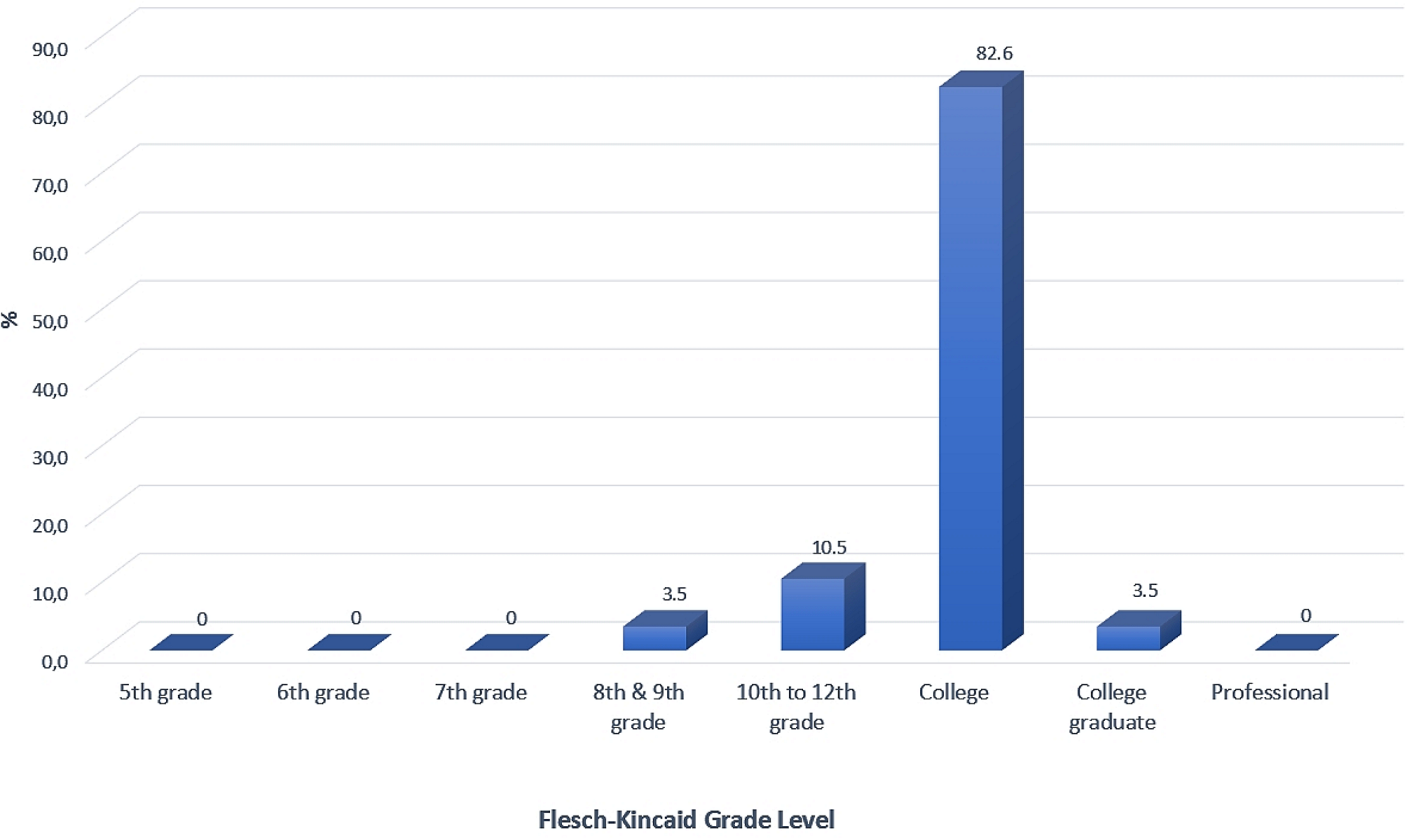
Flesch–Kincaid Grade Level (FKGL) of analyzed websites

Professional organization websites had the highest score for each DISCERN section (Table 1). The weakest aspect of all websites was that the risks of early orthodontic treatment options were not adequately mentioned (11th question). The information quality of most websites [72.1% (*n* = 59)] was very poor, while only 1.2% (*n* = 1) was considered excellent according to DISCERN criteria (Fig. 2). Orthodontist and multidisciplinary dental clinic websites had similar scores for all questions. The two groups had no significant difference regarding DISCERN scores (Table 2). Regardless of authorship, the readability of written information on all websites was quite difficult according to above-average FRES and FKGL scores (Figs. 3 and 4).

**Table 2:** Demographic information for all websites together and grouped according to authorship with comparison of scores for quality scores among groups

Parameters		Orthodontists (*n* = 69)				Private Dental Clinic (*n* = 13)				*p
		Mean (SD)	Min.-Max.	Median	25p-75p	Mean (SD)	Min.-Max.	Median	25p-75p	
**DISCERN**	**Section 1**	15.46 (2.45)	11–28	15	15–15	16.08(2.47)	13–21	15	14.5–18	0.421
	**Section 2**	11.41 (2.37)	8–24	11	11–11	11(1.47)	9–14	11	10-11.5	0.763
	**Total Mean**	26.88 (4.61)	19–52	26	26–26	27.23 (3.68)	22–33	26	24.5–30.5	0.352
	**Section 3**	2.12(0.4)	1–4	2	2–2	2.23 (0.44)	2–3	2	2-2.5	0.255
**FRES**		48.22 (6.49)	22.6–65	49.6	47.1–49.6	44.88 (10.14)	24.6–57.4	49.6	40.9–50.4	0.456
**FKGL**		11.52(1.59)	8.1–16.9	10.9	10.9-12.05	12.32(3.02)	8.6–19.6	11	10,9–13	0.366

SD, Standard Deviation; Min, Minimum; Max, Maximum; 25p, 25 Percentile; 75p, 75 Percentile; *Results of Mann-Whitney U test

**Fig. 5:**
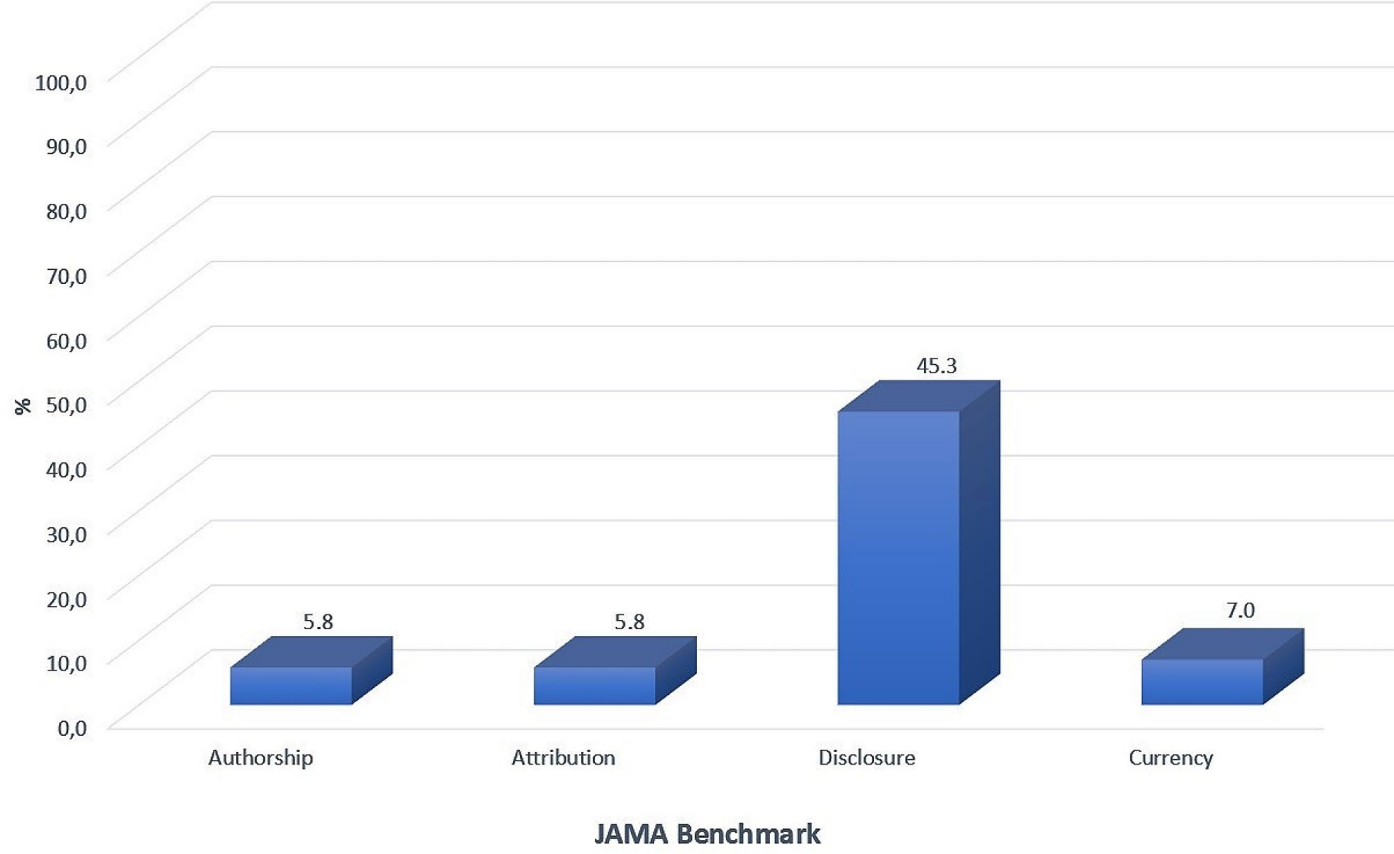
JAMA benchmark scores of analyzed websites

JAMA benchmark scores of websites were presented in Table 3. When websites were categorized according to authorship, no significant difference was found between the two groups regarding JAMA comparison criteria. The percent distribution of all websites according to JAMA is shown in Fig. 5. Forty-five and three-tenths percent of websites complied with disclosure, 7% with currency criterion, 5.8% with authorship criterion and 5.8% with attribution criterion. Only three websites met all JAMA criteria, and these websites belonged to professional organizations.

**Table 3:** Comparison of JAMA Benchmark scores among the groups

JAMA Benchmarks		Orthodontists (*n* = 69)	Private Dental Clinic (*n* = 13)	Total (*n* = 82)	*P Value
Authorship	No	68	12	80	0.294
	Yes	1	1	2	
Attribution	No	67	13	80	1.000
	Yes	2	0	2	
Disclosure	No	43	4	47	0.064
	Yes	26	9	35	
Currency	No	67	13	80	1.000
	Yes	2	0	2	

^*^Results of Fisher’s Exact test

## Discussion

Early orthodontic treatment aims to decrease the need for future orthodontic treatment or facilitate and shorten the duration of orthodontic treatment in future periods [25]. Malocclusions or dental alignment problems that have occurred due to oral habits seen in individuals are treated in the early stages and these problems are prevented from turning into skeletal anomalies [26].

This study is the first to evaluate website information’s quality, reliability, and readability about early orthodontic treatments. The Internet is an easy-to-access information platform. 80% of Internet users who want to access medical information use online tools to access this information [27]. However, the quality of online sources is variable. Healthcare professionals should be aware of the quality of Internet content because patients’ interest in online health-related information increases [28]. It has been suggested that most of the information on the Internet is either incomplete or inaccurate mainly in dentistry [11, 17–19, 29–32]. These results also show that the need for the accuracy and reliability of the information on websites.

People want easy-to-access, useful and understandable information through online research [19]. However, the quality and accuracy of the information that could be derived from the Internet might not be that high. The information on the Internet is presented without any filtering. This situation requires the use of assessment tools for the accuracy and reliability of the information on the websites. So we used DISCERN, JAMA assessment criteria, and HONcode tools which were preferred in previous similar researches [11, 18, 19, 30–32].

DISCERN is a standard tool to evaluate the quality and reliability of website information on the Internet [11]. According to the DISCERN tool, 72.1% of the websites evaluated in the study were of very poor quality. This study and other studies on different topics performed by different authors have given similar findings [11, 18, 19, 29–32]. When the sources of information on websites were reviewed, no significant difference was found between orthodontists and multidisciplinary dental clinics.

According to the results of this study, the highest scores on the JAMA criteria were disclosure (45.3%) and currency (7%); and the lowest scores were authorship (5.8%) and attribution (5.8%). This result is similar to Olkun et al.‘s study about lingual orthodontics [11]. In McMorrow et al.‘s [29] study performed in 2016 on adult orthodontics currency score was 85% which is similar to our study; however, the authorship score was 65% which is different from this study.

The mean FRES score of all websites was 46.55. This value means the readability of articles submitted to websites is “fairly difficult”. In addition, the mean FGKL value of all websites is 11.32. FRES score is higher in orthodontists (48.22) than in multidisciplinary dental clinics (44.88); however, there was no statistically significant difference between groups. Similar results have been obtained from studies on different topics [29, 33]. Health information websites should be easily readable by people with low level of knowledge. Therefore, easily read online information may be more effective in informing and attracting potential patients for early orthodontic treatment.

None of the 86 websites evaluated in this study have the HONcode logo. This may be because website designers do not know about HONcode. Besides, HONcode is a paid application. Website owners may not want to pay this fee. In the study of Meade and Dreyer [30], in which they evaluated the quality of information on the Internet about orthodontic temporary anchorage devices, it was seen that only one of the 31 websites they examined had HONcode. Likewise, in Meade and Dreyer [31] study on ectopic canine teeth in 2022, only 3 of 77 websites had HONcode.

In accordance with Graf et al.‘s research [34], it has been posited that the substitution of doctor-patient communication with purely evidence-based information may not be feasible. Recognising the Internet as an important source of information is imperative, and health professionals should develop effective strategies to facilitate patient guidance within this vast resource. Nevertheless, in light of the evaluations conducted using assessment instruments, it is discerned that the quality of websites falls short of the requisite standards. Therefore, the communication between medical practitioners and patients via oral interaction is regarded as more dependable than the information derived from online sources.

Bavbek and Tuncer [19] assessed thirty-six websites, employing the Turkish language as the search language. The study found that despite the apparent variability in the quality of information on the Internet, the overall quality rating, as measured by the DISCERN tool, tends to be low or medium for the majority of websites. Oey and Livas [35] assessed hundred websites, employing the Dutch language as the search language. The mean FRES derived from the examination of information presented on websites within the study presented textual content characterized by a level of complexity, indicating difficulty in understanding.Research conducted in diverse linguistic contexts analogous to the present study has revealed parallels with our findings.

There are several limitations in our study, like any other previous health-related studies on the Internet. Web search was limited to English websites in the United States, and other languages ​​were excluded. Therefore, the results are only valid for a limited population. Information about early orthodontic treatments constantly changes and is updated as information and the content on the Internet constantly change. The content of websites and rankings in search engines can change over time. This change may create differences in terms of the quality and readability of websites. The JAMA and DISCERN tools are based on subjective quality measurements. So, it does not seem possible for subjective bias to be completely eliminated. The JAMA comparison criteria are not always comprehensive or up-to-date. It was developed in 1999, and some of the criteria may not be relevant to the current state of medical information on the Internet. Another limitation of this research is that the reliability of the displays of the HONcode logo has been questioned [36]. Additionally, our study was unable to clarify the misleading use of the HONcode logo. The limitation in terms of readability is that the Flesch Reading Ease Score and Flesch-Kincaid Grade Level Score are only two measures of readability. The FRES and FKGL could not debate other factors that can affect readability, such as the use of jargon or technical terms.

## Conclusions

The information on early orthodontic treatment on websites is generally of low quality. Additionally, the readability level of website content is fairly hard according to recommended minimum readability level. Creating content by taking into account the quality assessment tools of websites containing information about early orthodontic treatments will be a great advantage for both lay persons with weak literature knowledge and professionals.

## Data Availability

The datasets used and analyzed during the current study are available from the corresponding author on reasonable request.
